# Syndecans and Their Synstatins: Targeting an Organizer of Receptor Tyrosine Kinase Signaling at the Cell-Matrix Interface

**DOI:** 10.3389/fonc.2021.775349

**Published:** 2021-10-27

**Authors:** Alan C. Rapraeger

**Affiliations:** Department of Human Oncology, School of Medicine and Public Health, University of Wisconsin-Madison, Madison, WI, United States

**Keywords:** EGFR, IGF-1R, CXCR4, VEGFR2, synstatin, integrin signaling, ASK-1

## Abstract

Receptor tyrosine kinases (RTKs) and integrin matrix receptors have well-established roles in tumor cell proliferation, invasion and survival, often functioning in a coordinated fashion at sites of cell-matrix adhesion. Central to this coordination are syndecans, another class of matrix receptor, that organize RTKs and integrins into functional units, relying on docking motifs in the syndecan extracellular domains to capture and localize RTKs (e.g., EGFR, IGF-1R, VEGFR2, HER2) and integrins (e.g., αvβ3, αvβ5, α4β1, α3β1, α6β4) to sites of adhesion. Peptide mimetics of the docking motifs in the syndecans, called “synstatins”, prevent assembly of these receptor complexes, block their signaling activities and are highly effective against tumor cell invasion and survival and angiogenesis. This review describes our current understanding of these four syndecan-coupled mechanisms and their inhibitory synstatins (SSTN_IGF1R_, SSTN_VEGFR2_, SSTN_VLA-4_, SSTN_EGFR_ and SSTN_HER2_).

## Introduction

The growth and progression of tumors is influenced by the tumor microenvironment, including growth factors, cytokines, and components of the extracellular matrix produced by the tumor stroma and the tumors themselves. Receptors on the surface of the tumor cells, of which growth factor receptor tyrosine kinases (RTKs) and integrins are prominent, mediate the response of the tumor to these factors, including enhanced proliferation, survival, metastasis, and resistance to therapeutic drugs. Effective RTK signaling often requires coordination with integrins and RTK activation in turn serves to potentiate integrin signaling (1–4). Integrins are cell-cell and cell-matrix receptors composed of α and β subunits that assemble into 22 distinct receptors with individual signaling and ligand specificity. Their activation is promoted by signaling from inside the cell (e.g., inside-out signaling), often from RTKs, leading to stable adhesion. In addition, they respond to ligands in the tumor microenvironment to activate signaling (outside-in signaling) through cytoplasmic effectors such as FAK, paxillin, Src kinases, and others, often coordinated with RTK signaling (1). It is well established, for example, that integrin-mediated matrix adhesion is required for growth factor-stimulated cell cycle progression [reviewed in (5, 6)]. This extends to the tumor microenvironment as well, where RTKs and integrins function on endothelial cells, stromal cells and immune cells participating in vascular angiogenesis, lymphangiogenesis, and inflammation in support of tumor growth (7). A number of examples have now emerged to suggest that this coordinated signaling requires the RTK and integrin to be assembled into a functional unit [see (8, 9)]. A well-described example involves the α6β4 integrin. Usually found in resting epithelia and endothelia as the central adhesion receptor in highly stable hemidesmosomes, the α6β4 integrin is converted to a signaling scaffold during wound healing or tumorigenesis when phosphorylated by serine-threonine kinases activated downstream of RTKs (10). The free integrin associates with RTKs (e.g., EGFR, HER2, c-Met, Ron kinase), resulting in phosphorylation of tyrosines in the β4 integrin cytoplasmic domain and activation of signaling effectors that drive proliferation, survival and invasion of the tumor cells (11–15). Another example is the interdependence of the αvβ3 integrin and VEGFR2, an example in which upregulated expression and activation of the αvβ3 integrin is observed on endothelial cells in response to the induction of angiogenesis by VEGF (16–18). When activated, these two receptors co-immunoprecipitate as a functional complex from the endothelial cells, and loss or prevention of activation of either receptor negatively impacts the activation of the other (16–18). The αvβ3 integrin also engages in coordinated signaling with the type 1 insulin-like growth factor receptor (IGF-1R) in smooth muscle cells where IGF-1R is recruited to active αvβ3 integrin, which attenuates the IGF-1R signal *via* SHP2 phosphatase localized to the integrin (19, 20). Despite these and other examples, however, the means by which the RTKs and integrins interact and whether or not these interactions are regulated, often remains unknown. Work over the past decade has identified the syndecans as potential organizers of these signaling units. This review will summarize several examples in which docking sites in the extracellular domains of syndecans serve to organize and regulate the signaling of RTKs and integrins.

## An Organizer Role for Syndecans

The syndecans are a family of four cell surface heparan sulfate (HS) proteoglycans, united by their highly homologous transmembrane and cytoplasmic domains, and their display of 2-3 HS glycosaminoglycan chains at the distal tips of their extracellular domains. These conserved features have attracted the attention of investigators attempting to understand syndecan functions [see reviews (21–30)]. In brief, the HS chains endow the syndecans with modulatory roles in numerous processes by engaging “heparin binding domains” in a variety of ligands, including growth factors (FGFs, VEGFs, PDGFs, HGF, EGFs, etc.), proteases and protease inhibitors, and essentially all extracellular matrix (ECM) ligands. Conserved alanine and glycine repeats interrupt their bulky hydrophobic membrane-spanning domains to promote syndecan homo- and heterodimerization, suggesting potential interactions with other membrane receptors as well. Exactly conserved C1 and C2 motifs found in their cytoplasmic domains bind to FERM- and PDZ-domain proteins and work in conjunction with syndecan-type-specific variable (V) domains to promote cytoplasmic activities; these range from chaperoning integrins and other receptors during endocytic and intracellular trafficking to signaling within focal adhesions. Less attention, however, has focused on their extracellular protein domains, which, apart from the conserved motifs that encode HS attachment, share little or no homology across the syndecan family. Emerging work now shows that these domains may be key to a central syndecan function, namely, acting as a “signaling organizer” at the cell surface by providing docking sites for other plasma membrane receptors, especially integrins and RTKs with well-known roles in cancer, that are highly dependent on the syndecan for their activation and signaling.

## Coupling of IGF-1R to the αvβ3 or αvβ5 Integrin

The αvβ3 and αvβ5 integrins have been shown in now classical studies from the Cheresh group to play essential roles in vascular angiogenesis stimulated by fibroblast growth factor-2 (FGF-2) and vascular endothelial growth factor (VEGF) (31). These integrins also have other tumor-promoting activities, including displaying upregulated expression in a variety of tumor cells, having a role in the differentiation of tumor progenitor cells and being essential for the differentiation and bone-eroding activity of osteoclasts triggered by tumors homing to the bone marrow (7). The type 1 insulin like growth factor receptor (IGF-1R) is widely expressed in normal tissues and has important roles in prenatal and postnatal organ growth, with key roles in cell cycle progression and cell survival signaling (32). Pups of IGF-1R null mice are born with severe organ hypoplasia and are less than half of normal size (33). These roles are equally, if not more, striking in cancer. Fibroblasts derived from IGF-1R null mice resist transformation by most oncogenes, and tumor growth in mice is blocked by IGF-1R siRNAs that otherwise have little or no effect on normal tissues (34, 35).

**Figure 1:**
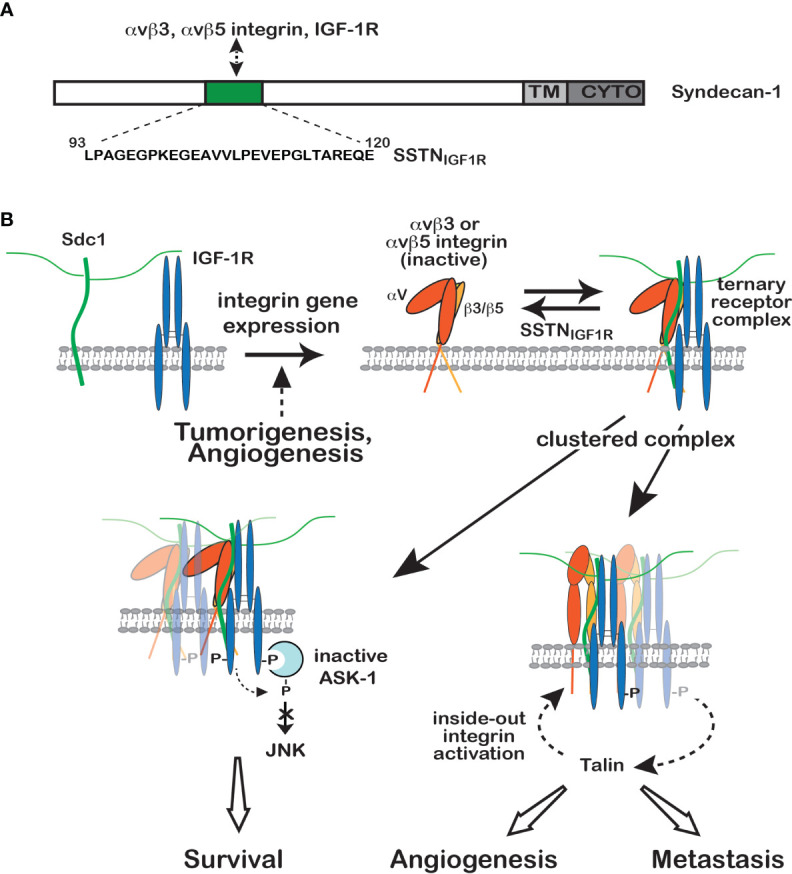
Regulation of IGF-1R signaling by Sdc1. **(A)** Model depicting site of IGF-1R capture, along with either the αvβ3 or αvβ5 integrin, in the extracellular domain of human Sdc1. A peptide mimetic of this site, SSTN_IGF1R_, competitively blocks receptor binding. **(B)** IGF-1R is not engaged with Sdc1 in nontransformed cells, typically because they lack the αvβ3 and αvβ5 integrins (top left). But integrin expression, usually in response to malignant transformation or activation of endothelial cells during angiogenesis, results in integrin docking to the extracellular domain of Sdc1, which is followed by IGF-1R capture at the same docking site (top right). Once formed, constitutive or matrix-induced clustering of the ternary receptor complex activates IGF-1R by autophosphorylation that does not require IGF-1. Activated endothelial cells or tumor cells bearing the ternary receptor complex rely on the syndecan-activated IGF-1R to phosphorylate and suppress the activity of Apoptosis-Signal regulating Kinase-1 (ASK-1) engaged with the IGF-1R cytoplasmic domain (bottom left), preventing ASK-1-mediated activation of Jun-N-terminal Kinase (JNK) and blocking entry into apoptosis, thus promoting tumor cell survival. In a second activity (bottom right), downstream signaling from IGF-1R activates the αvβ3 or αvβ5 integrin *via* an inside-out signaling pathway that targets the integrin-activating protein Talin, resulting in endothelial or tumor cell motility during the onset of angiogenesis or tumor cell invasion. SSTN_IGF1R_ competitively disrupts the ternary receptor complex, preventing integrin activation and removing the constitutive suppression of ASK-1. Neither activity can be rescued by IGF-1 when the receptor complex is disrupted by SSTN_IGF1R_, emphasizing the singular role played by the syndecan in this IGF-1R signaling mechanism (see discussion in text, references (27, 39–41) and references therein.

An involvement of IGF-1R in cell-matrix adhesion came to light when the Clemmons group demonstrated an important co-regulatory interaction between the αvβ3 integrin, the integrin-associated protein (IAP) and IGF-1R in smooth muscle cells, in which IGF1 stimulates activation of the integrin and the integrin attenuates IGF-1R signaling by recruitment of the phosphatase SHP-2 (19, 20, 36–38). Subsequent studies focusing largely on cancer mechanisms have shown that the activities of the αvβ3 and αvβ5 integrins and IGF-1R are linked and regulated by syndecan-1 (Sdc1). The αvβ3 and αvβ5 integrins are well-studied integrins that are upregulated in the tumor microvasculature and in many tumors as well, where they are likely to engage provisional matrix consisting of vitronectin, fibronectin, osteopontin, fibrinogen and von Willebrand factor in the tumor microenvironment (7). A pre-assembled complex consisting of Sdc1, inactive αvβ3 or αvβ5 integrin and inactive IGF-1R is found in fibroblasts, breast cancer and other carcinomas, multiple myeloma and activated vascular endothelial cells undergoing pathological angiogenesis (39–42) (**Figure 1**) and is likely found in many cancers. Clustering of this ternary receptor complex, occurring when Sdc1 in adherent cells engages the vast polyvalent array of heparin-binding domains presented by proteins in the basement membrane and stromal ECM, or mimicked by plating cells on Sdc1-specific antibody, leads to ligand-independent activation of the IGF-1R, which initiates inside-out signaling through talin to activate the integrin (41) (**Figure 1B**). Syndecan mutagenesis studies, together with competitive inhibition of IGF-1R or integrin activation by recombinant Sdc1 ectodomain or peptides derived from the Sdc1 ectodomain, localizes the integrin/IGF-1R docking site to amino acids 93-120 in the extracellular domain of the membrane-anchored human Sdc1 (27, 39) (**Figure 1A**). A peptide mimetic of this site, originally called “synstatin” (SSTN) and now referred to as “SSTN_IGF1R_” to distinguish it from other synstatins subsequently identified against other RTKs, directly binds the integrins and IGF-1R and prevents their interaction with the syndecan (**Figure 1A**). This blocks the autophosphorylation of IGF-1R, even in the presence of IGF-1 (40), and prevents activation of the integrin. *In vitro* binding experiments using purified IGF-1R, integrin and recombinant Sdc1 extracellular domain show that the ternary complex can be formed *in vitro* from these three components alone; but, whereas the integrin alone is readily captured by Sdc1, IGF-1R binds only after the integrin is engaged by the syndecan, suggesting that IGF-1R docks to an interface comprised of Sdc1 together with the integrin (41). This feature of the capture mechanism serves to restrict this unique regulation of IGF-1R signaling to select cell types that express all three receptors, typically tumor cells and endothelial cells undergoing pathological angiogenesis. Whether there are additional membrane components expressed in tumor cells that stimulate the assembly of this receptor complex ranging from protein partners such as tetraspannins to specialized lipid rafts has not been explored.

Immunoprecipitation studies suggest that the majority, if not all, of the IGF-1R in tumor cells is associated with Sdc1, potentially making SSTN_IGF1R_ a highly specific cancer therapy. It resists proteolytic degradation in plasma that usually leads to the rapid demise of most peptides *in vivo*, and is cleared only slowly from the circulation, displaying a t½ of 27 hr in mice (40). It is highly effective against tumor xenografts, which are reduced in size over 10-fold in mice treated with 0.365 mg/kg/day of the peptide, sufficient to reach a concentration of 3 μM in the blood (39, 40). Restriction of the mechanism to tumors and pathological angiogenesis and its apparent absence in normal tissues suggests that SSTN_IGF1R_ may have little impact, if any, on normal metabolism and low toxicity to normal organs. Conventional therapeutic approaches such as IGF-1R kinase inhibitors also target the insulin receptor and the IGF-1R-feedback loop in the hypothalmus, leading to increased insulin and IGF1 levels in the plasma that are thought to stimulate rather than disrupt tumorigenesis (43). IGF-1R-blocking antibodies developed as potential therapeutics also disrupt the feedback loop and fail to disrupt IGF1R-coupled to Sdc1, which is IGF1-independent (43).

Examination of SSTN-treated tumors demonstrates that not only are they reduced in size, but they display a 10-fold reduction in vascularization as well. This is due to SSTN_IGF1R_’s activity against both the tumor cell, in which it not only blocks migration but also activates apoptosis, and against activated endothelial cells engaged in tumor-induced angiogenesis. The peptide blocks αvβ3-mediated migration of vascular endothelial cells *in vitro* with an IC_50_ of ca. 300 nM and displays similar activity against FGF-induced corneal angiogenesis *in vivo* when delivered systemically *via* Alzet pump (39). This traces to the dependence of VEGF signaling on the αvβ3 integrin. A clear example of this dependence is the defective VEGFR2 activation and blocked angiogenesis observed in genetically-engineered mice expressing the β3^Y747F/Y757F^ integrin mutant, which prevents αvβ3 integrin activation and signaling (16, 17). In similar fashion, treatment of endothelial cells with SSTN_IGF1R_ not only blocks αvβ3 activation, but also prevents VEGFR2 activation by VEGF (44). The Sdc1-, IGF-1R- and integrin-dependent regulation of VEGFR2 activation depends on cell-cell contact mediated by VE-cadherin, long known to be a regulator of VEGFR2 activation and angiogenesis (45, 46), linking these disparate mechanisms. This linkage appears to be complex and remains largely unknown, but depends in some manner on the clustering of VE-cadherin (44) and activation of Src (17), ostensibly to phosphorylate tyrosines Y747 and Y757 in the β3 integrin cytoplasmic domain (17). The activation of VEGFR2 by VEGF is blocked by VE-cadherin blocking antibodies, but VEGFR2 activation is restored if the blocking antibodies engaged with the cadherin are artificially clustered, mimicking VE-cadherin clustering that occurs during cell-cell adhesion (44). In step-wise fashion, this causes IGF-1R autophosphorylation that is nonetheless dependent on Src and is prevented by SSTN_IGF1R_, followed by downstream activation of VEGFR2 (44).

## Sdc1-Coupled IGF-1R in Tumor Cell Survival

Perhaps the greatest impact of Sdc1-coupled IGF-1R on tumorigenesis is its role in tumor survival, revealed in myeloma cells that express high levels of Sdc1 (CD138) and constitutively active Sdc1-coupled IGF-1R. SSTN_IGF1R_ induces the rapid activation of Jun-N-terminal kinase (JNK)/stress-activated protein kinase (SAPK) in myeloma cells, and p38MAPK as well in activated endothelial cells and carcinoma cells, two stress-activated MAPKs capable of initiating apoptosis in response to genotoxic and metabolic stressors common in cancer (40). A key upstream activator in myeloma appears to be Apoptosis-signal Regulating Kinase-1 (ASK-1), a MAP3K with critical roles in plasma cell and myeloma cell survival (47). ASK-1 binds the cytoplasmic domain of IGF-1R and is maintained in an inactive state by IGF-1R-mediated tyrosine phosphorylation, as well as by serine-threonine phosphorylation carried out by other enzymes activated downstream of IGF-1R (48, 49). However, inhibition of IGF-1R by SSTN_IGF1R_ prevents these inhibitory phosphorylation events, allowing ASK-1 autoactivation, its subsequent activation of JNK/SAPK, and entry of myeloma cells into apoptosis (40) (**Figure 1B**). It is of interest to note that although SSTN_IGF1R_ inhibits both IGF-1R and its downstream activation of the αvβ3 or αvβ5 integrin in endothelial and carcinoma cells, the integrin is not active in the myeloma cells, suggesting that it is IGF-1R alone that is required for the survival signaling (40). The signaling pathway downstream of IGF-1R that activates talin and thus the αvβ3 or αvβ5 integrin remains to be identified and the question remains as to whether it is present but inactive in myeloma cells, or if a critical component of the pathway is lacking altogether. Also unique to the myeloma cells compared to carcinoma or endothelial cells is that IGF-1R in the pre-assembled Sdc1-coupled ternary receptor complex is constitutively active (40); it is not dependent on cell adhesion or IGF-1, although exogenous IGF1 stimulates increased levels of IGF-1R activation. But even the IGF1-mediated activation is strictly dependent on the interaction of IGF-1R with the syndecan and is blocked by SSTN_IGF1R_ (40).

Several key questions about this activation mechanism remain unanswered, including how clustering of Sdc1 activates IGF1-R independent of IGF1 ligand, how SSTN_IGF1R_ blocks IGF1 stimulation of IGF-1R in myeloma and whether SSTN_IGF1R_ blocks IGF1-induced IGF-1R activation in tumors other than myeloma. IGF-1R is a dimeric receptor composed of α and β subunits that relies on tyrosine kinase phosphorylation to recruit adaptor proteins (e.g., IRS-1, IRS-2, Shc) that mediate signaling leading to cell proliferation, cell migration and apoptosis (7). High resolution structural modeling suggests that the receptor is auto-inhibited due to wide separation of its cytoplasmic kinase domains (50). Binding of a single IGF1 monomer relieves these constraints and fosters a pseudoligand interaction between the two extracellular domains that re-positions the cytoplasmic kinase domains to undergo transphosphorylation (50). In the case of IGF-1R activation by Sdc1, it is clear that clustering of the syndecan must in some manner reproduce this positioning of the kinase domains. One scenario suggests that IGF-1R is optimally oriented when complexed with Sdc1 and αvβ3 or αvβ5 integrin to favor autophosphorylation in *trans* between the kinase domains of distinct IGF-1Rs when clustered by the syndecan. A less likely scenario envisions interactions between the IGF-1R extracellular domains promoted by clustering that mimic ligand binding and thus re-orient and activate the kinase domains in a single receptor similar to IGF1 binding.

## Sdc1 as an Organizer of VLA-4/VEGFR2-Mediated Cell Polarity and Invasion

The α4β1 integrin, known as VLA-4 (very late antigen-4) on leukocytes, modulates the recruitment of leukocytes during immunity and autoimmune diseases, the dissemination of stem cell precursors into the blood, and the invasive phenotype of blood cell cancers (51). The integrin recognizes several ligands, but most prominently vascular cell adhesion molecule-1 (VCAM-1), which is largely expressed on vascular endothelial cells and stromal cells in the bone marrow, and the stromal matrix macromolecule fibronectin, in which it recognizes a VLA-4-specific PVD binding motif in the CS-1 domain (52). Engagement of these ligands regulates the extravasation of leukocytes through the blood vessel wall and their migration within the interstitial matrix and has been shown to have a similar role on some tumor cells (reviewed in (51). VLA-4 is also expressed on vascular and lymphatic endothelial cells where it plays a role during angiogenesis and polarized re-orientation of endothelial cells in response to blood flow (53, 54). A critical step in these activities is the binding of the scaffolding protein paxillin to the cytoplasmic domain of the α4 integrin subunit, which localizes paxillin-bound signaling effectors to the integrin, including focal adhesion kinase (FAK), proline-rich tyrosine kinase-2 (Pyk2) and G-protein coupled receptor kinase interacting protein-1 (GIT1), an Arf-6 GAP (55–58). GIT1 prevents the local activation of Arf6 and its downstream target Rac-1, the latter necessary for lamellipodium formation in response to VLA-4-mediated adhesion. In contrast, FAK and Pyk2 provide downstream transregulatory signaling leading to activation of leucocyte functional antigen-1 (LFA-1, also known as the αLβ2 integrin), a second integrin important in leukocyte extravasation, shifting the cell invasion behavior to this integrin (57). However, phosphorylation of the α4 cytoplasmic domain on S988 by PKA, which occurs only at the site destined to be the leading edge of the cell, releases the block to Rac1 activation by displacing paxillin from the integrin, and induces VLA-4-mediated, rather than LFA-1-mediated, generation of an active lamellipodium and polarized migration of the cell (54, 59). But how PKA is activated, and how it is localized to the integrin in order to carry out this phosphorylation, has remained unknown.

**Figure 2:**
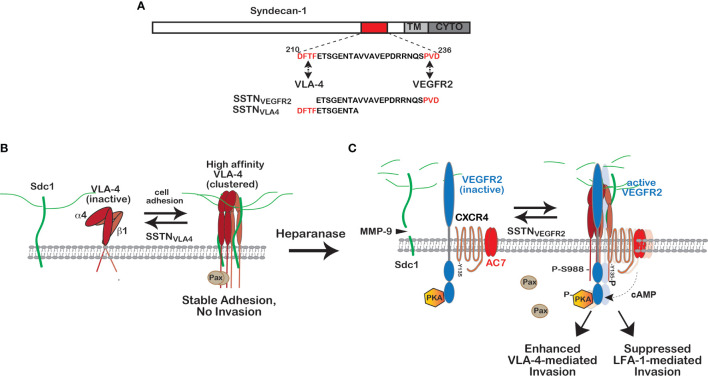
Regulation of VLA-4 activation and polarized cell invasion by Sdc1. **(A)** *VLA-4 and VEGFR2 docking sites in Sdc1.* Juxtamembrane sites in the extracellular domain of human Sdc1 (DFTF and PVD) responsible for engaging very late antigen-4 (VLA-4, α4β1 integrin) and vascular endothelial growth factor receptor-2 (VEGFR2), respectively. Synstatin peptides containing one, but not both of these sites (e.g., SSTN_VLA-4_ or SSTN_VEGFR2_), prevent VLA-4 or VEGFR2 capture by the syndecan and disrupt signaling that relies on the co-capture of both receptors. **(B)** *Regulation of high affinity VLA-4 adhesion by Sdc1.* VLA-4 undergoes rapid activation when engaging ligand, involving a conformation change and clustering (avidity modulation) to increase binding affinity. On cells expressing Sdc1 (e.g., myeloma cells, vascular endothelial cells, melanoma, Jurkat-T cells), this activation is blocked by preventing VLA-4 docking with Sdc1 using SSTN_VLA-4_ or mutating the DFTF motif in the syndecan. The mechanism underlying this dependence on the syndecan remains under investigation. **(C)** *Role of heparanase and shed Sdc1 in polarized cell invasion*. The heparan-sulfate-degrading enzyme heparanase is a known tumor promoter and enhancer of leukocyte recruitment during inflammation. Trimming of the HS chains on Sdc1 exposes its core protein to cleavage by matrix-metalloproteinase-9 (MMP-9), releasing the syndecan ectodomain. The shed syndecan couples an inactive receptor complex consisting of VEGFR2 with inactive protein kinase A (PKA) attached to its cytoplasmic domain, the cytokine receptor CXCR4 and adenylate cyclase 7 (AC7) to the clustered integrin, causing VEGF-independent activation of VEGFR2; VEGFR2 phosphorylates CXCR4 on Y135, activating its Gα_i_βγ GTPase and the Gα_i_-dependent AC7. Local generation of cAMP leads to activation of protein kinase-A (PKA) engaged with the VEGFR2 cytoplasmic domain and phosphorylation of the α4-integrin cytoplasmic domain on serine 988. This displaces the Rac-inhibitory paxillin from the integrin, causing polarized invasion of VLA-4-dependent immune cells, typically tumor supporting cells such a macrophages, MDSCs and others, as well as myeloma cells. Displacement of paxillin also inhibits the cross-talk between VLA-4 and LFA-1 necessary for LFA-1-mediated migration that characterizes tumor suppressor cells, such as NK and cytotoxic T cells. Either prevention of integrin activation by SSTN_VLA-4_ or VEGFR2-coupling to the integrin by SSTN_VEGFR2_ serves to block these processes [see discussion in text, references (60, 64) and references therein].

A recent clue to the activation mechanism comes from the study of multiple myeloma, a disease in which malignant plasma cells extravasate throughout the bone marrow of affected patients, and a unique response of the cells when CD138 (syndecan-1) is targeted by heparanase (HPSE), an HS-degrading endoglucuronidase (60). Although circulating B lymphocytes reportedly express low levels of syndecans, if any, the expression of Sdc1 is greatly upregulated in plasma cells and their malignant counterparts. Poor prognosis in the disease accompanies those tumors that express high levels of HPSE, which trims the HS chains of Sdc1 and causes shedding of high levels of Sdc1 into the bone marrow microenvironment (61–63). Using human CAG myeloma cells expressing low versus high levels of HPSE as a test system, it is observed that high HPSE expression promotes an invasive phenotype that depends on VLA-4; VLA-4 is localized to the leading edge when the cells are plated on fibronectin or VCAM-1, and is responsible for cell invasion through filters coated with these VLA-4 ligands (60). Several lines of evidence trace the polarization mechanism to Sdc1 ectodomain that has been shed from the cell surface. First, polarization is blocked by MMP-9 inhibitors that prevent shedding of the syndecan. Second, polarization can be rescued when shedding is blocked if conditioned medium containing shed Sdc1 is added to the cells, but not if Sdc1 is immunodepleted from the medium. Third, the invasive phenotype on VLA-4 ligands can be stimulated by exogenous recombinant human Sdc1 ectodomain alone in the absence of HPSE, or a peptide representing a putative active site contained within amino acids 210-236 of the recombinant protein (**Figure 2A**). An asp-phe-thr-phe (DFTF) motif at the N-terminus of this peptide represents a site in the Sdc1 ectodomain that is essential for binding VLA-4 and a pro-val-asp (PVD) sequence at its C-terminus mimics a motif in Sdc1 that captures vascular endothelial cell growth factor receptor-2 (VEGFR2) (60) (**Figures 2A** **, B**).

The proposed mechanism that emerges from these studies is as follows. Engagement of VLA-4 with its ligands causes its rapid activation and clustering (avidity modulation), an initial first step designed to rapidly strengthen its affinity for VCAM-1 on the blood vessel wall during leukocyte activation and extravasation from the blood stream (58). In myeloma, this initial step requires association of VLA-4 with membrane-bound Sdc1 *via* its DFTF motif (60) (**Figure 2B**), although whether this docking plays a role in the integrin’s conformational change that promotes ligand engagement, or in strengthening the adhesion by avidity modulation, or in some other step, is not yet clear. This step is prevented by a syndecan-derived peptide containing the DFTF motif (SSTN_VLA4_) but lacking the PVD motif necessary for capturing VEGFR2 (60) (**Figure 2C**). Next, shedding of Sdc1, induced by HPSE-mediated trimming of its HS chains and carried out by MMP-9, releases Sdc1 from its membrane domain and allows it to engage VEGFR2, which requires the PVD motif in the syndecan (**Figure 2C**). The result is coupling of VEGFR2 to the clustered VLA-4 by shed Sdc1, which in turn clusters and activates VEGFR2 *via* autophosphorylation, and induces the polarized invasion of the cells. No VEGF is required and VEGFR2 blocking antibodies fail to disrupt this mechanism (60).

The next important step in the understanding of this mechanism is the target of VEGFR2 phosphorylation that activates and localizes PKA to the integrin, where it carries out the phosphorylation of S988 on the α4 subunit. Cell staining demonstrates that shed Sdc1 causes VLA-4 to re-localize from the lagging edge to the leading edge of myeloma cells where it localizes with the shed Sdc1 and VEGFR2 when cells are plated on VLA-4 ligands (60). Recent work now shows that VEGFR2 is preassembled into a complex with PKA, the cytokine receptor CXCR4, and adenylate cyclase 7 (AC7) (64) (**Figure 2C**). The target of the VEGFR2 kinase that is activated by clustering when engaged with the shed Sdc1 is tyrosine 135 (Y135) within an asparagine-arginine-tyrosine (DRY) microswitch in cytoplasmic loop 2 of CXCR4, part of an activation switch present throughout the broad superfamily of G-coupled protein receptors (65). Although this tyrosine is highly conserved in the superfamily, there was little prior evidence to support a direct role in G-protein activation until to this demonstration of CXCR4 activation by VEGFR2 phosphorylation. Activation of VLA-4 can also occur if CXCR4 in this complex is activated by its ligand, CXCL12, but this still requires linkage of the complex to VLA-4 by shed Sdc1 (64). Activation of CXCR4 and its Gα_i_βγ heterotrimeric G-protein activates AC7, one of a small number of adenylate cyclases that are activated by Gα_i_-containing G-proteins, resulting in increased cAMP levels that locally activate PKA-mediated phosphorylation of Y988 in VLA-4 (64).

This VLA-4 activation mechanism is blocked by syndecan-derived peptides that contain the PVD motif (SSTN_VEGFR2_) and lack the DFTF motif, thus binding VEGFR2 and competitively preventing it from engaging shed Sdc1 and docking with the integrin (60). These two SSTN peptides provide new tools to either block VLA4-mediated adhesion altogether (SSTN_VLA4_) or to specifically target VLA-4 actively engaged in cell invasion (SSTN_VEGFR2_). It is envisioned that in myeloma this mechanism plays a major role in the extravasation of myeloma throughout the bone marrow by engaging VCAM-1 expressed by the vascular endothelium of bone marrow capillaries and VCAM-1 and fibronectin that are richly expressed in the bone marrow stroma. Although initially discovered in HPSE-overexpressing myeloma cells, this mechanism is also operative on vascular endothelial cells, in which VEGFR2 and the α4β1 integrin are commonly expressed (60). VEGFR2 has been shown previously to associate with Sdc1 in myeloma-derived vascular endothelial cells (66). In addition, the short-chain (scFv) recombinant human antibody OC-46F2, which binds to a juxtamembrane site in the Sdc1 ectodomain that overlaps with the VEGFR2 binding motif, disrupts angiogenesis and melanoma xenograft growth in NOD-SCID mice (67).

Further insight into this mechanism and its role in tumorigenesis emerges from an understanding of the role played by α4-S988 phosphorylation during the transregulation that occurs between VLA-4 and LFA-1 (the αLβ2 integrin), the latter utilized for invasion by tumor suppressing cells such as natural killer cells and cytotoxic T-cells (57, 68, 69). LFA-1 activity requires co-signaling from VLA-4 that depends on paxillin, PyK2 and FAK engagement with VLA-4 (57, 58, 70). Thus, phosphorylation of Y988 that displaces paxillin and enhances VLA-4-mediated invasion serves to suppress LFA-1-mediated invasion. This is elegantly shown by the increased LFA-1 activity of cytotoxic leukocytes bearing phosphorylation-resistant α4-S988A integrin; these cells appear to invade the tumor microenvironment more readily, leading to enhanced destruction of melanoma xenografts (71). It has been shown more recently that mouse NK and cytotoxic T-cells rely on Sdc1 as a regulator of this mechanism. NK and T-cells express Sdc1, VEGFR2, CXCR4, AC7 and HPSE and their LFA-1-mediated migration in *in vitro* invasion assays is dramatically increased by SSTN_VEGFR2_ while at the same time the peptide inhibits the VLA-4-mediated invasion of myeloma cells (64). This brings into focus the protumorigenic role of heparanase in myeloma and other tumors (72, 73). Myeloma patients with advanced disease are known to express high levels of heparanase and to have high levels of shed Sdc1 in the tumor microenviroment and the blood (74–76). By engaging VLA-4 and VEGFR2 on the myeloma cells and tumor suppressor cells in the tumor microenvironment, the shed syndecan is likely to promote VLA-4-mediated extravasation of myeloma cells in and out of the bone marrow and to extramedullary organs. At the same time, the LFA-1-mediated invasion of NK and cytotoxic T cells is suppressed by the Sdc1 shed into the tumor microenvironment, providing a protected environment for growth of the tumor (71, 77).

## Coupling of EGFR and HER2 to the α3β1 and α6β4 Integrins by Syndecans

**Figure 3:**
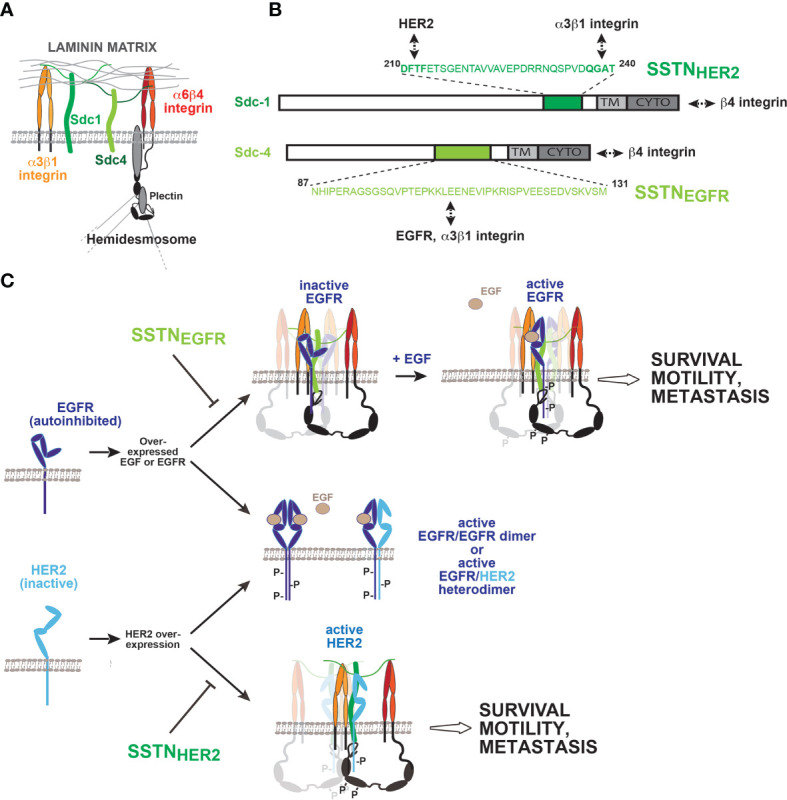
Coupling of EGFR family members to integrins by Sdc1 and Sdc4 regulates wound healing, tumor cell invasion and survival. **(A)** *Syndecans and integrins on quiescent epithelial or endothelial cells.* The laminin-332 (LN332) binding α3β1 and α6β4 integrin, along with Sdc1 and Sdc4 *via* their heparan sulfate chains, mediate binding to LN332-rich basement membrane. The long (>1,000 amino acids) cytoplasmic domain of the β4 integrin subunit engages the plectrin and BP180 scaffolding proteins, which engage the intermediate filament network and help stabilize the incorporation of the α6β4 integrin into hemidesmosomes. **(B)** *Sites of EGFR, HER2 and integrin capture in Sdc1 and Sdc4.* The multifunctional juxtamembrane site in Sdc1 captures HER2 and the α3β1 integrin. Multiple binding interactions occur throughout the sequence; nonetheless, the DFTF motif that is also essential for binding VLA-4 plays a prominent role in HER2 binding, whereas α3β1 integrin capture is highly dependent on the QGAT motif. An analogous juxtamembrane site in Sdc4, which bears no homology to the site in Sdc1, captures EGFR and the α3β1 integrin, relying on multiple binding interactions throughout the sequence. Synstatin peptides based on these sites are highly selective for EGFR (SSTN_EGFR_) or HER2 (SSTN_HER2_) and displace α3β1 integrin only when coupled with the EGFR family member for which they are specific. In addition to these interactions, the C-termini of the syndecans engage the cytoplasmic C-terminus of the β4 integrin, which comprises the α6β4 integrin. This is also syndecan-type specific; mutations that disrupt Sdc1 binding having no effect on Sdc4 and *vice versa*. **(C)** *Formation and activation of syndecan-organized quaternary receptor complexes containing EGFR or HER2.* EGFR and HER2 are overexpressed in squamous cell carcinomas, along with EGF. EGFR depends on EGF for dimerization, and to relieve constraints in its extracellular domain that prevent dimerization. HER2 lacks these extracellular constraints but lacks a ligand to promote its dimerization. Relief of the inhibitory constraints in EGFR allows it to form active homodimers or active heterodimers with HER2. Activation of receptor tyrosine kinases causes the breakdown of hemidesmosomes, freeing the cytoplasmic domain of the β4 integrin to engage Sdc1 and Sdc4. Quaternary receptor complex is formed when EGFR and the α3β1 integrin, or HER2 and the α3β1 integrin assemble with their respective docking site on the syndecans as well. The kinases appear to be captured as monomers and rely on clustering of the complexes to sites of matrix adhesion to be activated. Simple clustering of the syndecan is sufficient to activate HER2, but EGFR also requires EGF, ostensibly to relieve the dimerization constraints in its extracellular domain. Several tyrosines in the β4 cytoplasmic domain become phosphorylated, and support signaling that leads to epithelial cell migration during wound healing, or invasion and survival signaling in tumor cells and endothelial cells engaged in pathological angiogenesis. Synstatin peptides specific for HER2 capture by Sdc1 (SSTN_HER2_) or EGFR capture by Sdc4 (SSTN_EGFR_) are highly specific inhibitors of these processes. [See discussion in text, references (81–83) and references therein].

Work from multiple laboratories has shown that the α6β4 integrin has a signaling role during epithelial wound healing, carcinoma invasion and survival (10). This contrasts with its role on quiescent cells, where it functions as the central adhesion receptor in hemidesmosomes found in epithelial and endothelial cell layers (78–80) (**Figure 3A**). Here, its uniquely long cytoplasmic domain (over 1,000 amino acids) is linked by plectin to the keratin cytoskeleton, and its extracellular domain engages laminin 332 (LN332, also known as laminin 5) in the basement membrane (84, 85). But activation of RTKs, of which EGFR, HER2, and HGFR (c-Met) have been clearly implicated, causes hemidesmosome breakdown, freeing the integrin to associate with these kinases and resulting in phosphorylation of tyrosines in its “signaling domain” that acts as a scaffold for binding Shc, IRS-1/2 and other mediators in order to activate cell proliferation, invasion and survival (11, 13, 86–92). Cell migration utilizing this integrin occurs on a polarized substratum of LN332 that the cells deposit as they migrate through cooperative signaling from the α6β4 and α3β1 integrin (93). LN332 expression is upregulated along with the β4 integrin in epidermal squamous cell carcinomas (94) and these and other carcinomas depend on it and the α3β1 and α6β4 integrins for their invasion and survival (reviewed by Marinkovich (95). Mouse tumor models expressing a β4 mutant lacking its “signaling domain” (β4^1355T^) have been instrumental in demonstrating its critical role in tumorigenesis and tumor-induced angiogenesis that depends on EGFR, HER2 or c-MET (12, 96, 97). Importantly for this review, two of these RTKs (EGFR and HER2) rely on syndecans to pair them with the α6β4 and α3β1 integrins to carry out these important signaling roles (81, 82).

Immunoprecipitation studies show that Sdc1 is assembled into a quaternary receptor complex with HER2, the α6β4 integrin and the laminin-binding α3β1 integrin (81–83) (**Figure 3C**). A homologous receptor complex containing α3β1 and α6β4 integrins, along with EGFR rather than HER2, assembles with Sdc4 rather than Sdc1 (**Figure 3C**) (81, 83). The fact that homologous, but clearly distinct, receptor complexes are organized by these two syndecans is explained by the finding that they depend on the two domains of the syndecans that set them apart as family members: their extracellular domain and the V region of their cytoplasmic domains. Sdc1 captures HER2 and the α3β1 integrin *via* a juxtamembrane “co-receptor” binding site in its extracellular domain (amino acids 210-240) that is unique to Sdc1 (**Figure 3B**). A peptide mimetic of this extracellular docking site (called SSTN_HER2_) prevents capture of HER2 and the integrin and blocks epithelial cell motility on LN332 in wound healing assays (**Figure 3C**) (83). Binding studies using recombinant HER2 and Sdc1 extracellular domain show that the binding is direct and is competed by the SSTN_HER2_ peptide. Interestingly, the juxtamembrane site in Sdc1 required for the assembly of this receptor complex overlaps extensively with the site required for Sdc1 to capture VLA-4 and VEGFR2. Indeed, the DFTF motif necessary for VLA-4 capture plays a role in HER2 binding as well. In contrast, however, the PVD motif required to bind VEGFR2 appears to have no role in binding either HER2 or α3β1 integrin; instead, a QGAT motif (amino acids 237-240) must be present (83).

In addition to this extracellular docking site, a five-amino acid motif (QEExYx-c) at the C-terminus of the short Sdc1 cytoplasmic domain, comprised partly of its syndecan-specific V region (.QE.) and the C2 region shared by all four syndecans (…EFYA-c) engages the extreme C-terminus of the β4 integrin cytoplasmic domain, presumably positioning the integrin tail at the plasma membrane and in close proximity to HER2, where it’s signaling domain is phosphorylated by Fyn downstream of activated HER2 (81, 82). Deletion of the C2 region of Sdc1 or the last 28 amino acids in the β4 integrin tail (Δ1728-1752), or mutation of R1733 within this sequence, abolishes the interaction, prevents tyrosine phosphorylation of the integrin and blocks epithelial cell migration on LN332 and breast carcinoma cell survival.

The functional assembly of the Sdc4-coupled receptor complex occurs in a similar manner, but utilizing Sdc4-specific protein sequences. Sdc4 also binds the extreme C-terminus of the β4 integrin but requires E1729 rather than the R1733 required by Sdc1; introducing an E1729A mutation into the β4 cytoplasmic domain prevents its association with Sdc4 but has no effect on its ability to engage Sdc1 (81). Similarly, the R1733A mutant that fails to bind Sdc1 maintains its interaction with Sdc4. These β4 integrin mutants act as dominant negative receptors in cultured cells by outcompeting the wt β4 subunit for a limiting supply of α6 integrin subunit when overexpressed. Integrin subunits containing the R1733A mutation fail to activate HER2-dependent cell migration that depends on Sdc1 yet trigger migration in response to EGF. The E1729A mutant integrin sustains HER2-dependent migration, but cells expressing this mutant fail to migrate when stimulated with EGF.

Analogous to Sdc1, an extracellular, juxtamembrane site in Sdc4 consisting of amino acids 87-131 is responsible for capturing the α3β1 integrin and EGFR (**Figure 3A**) (83). A peptide mimetic of this site (SSTN_EGFR_) competes for the interaction and displaces EGFR and α3β1 integrin from the remaining Sdc4-α6β4 integrin duplex. As observed for Sdc1 and HER2, the interaction between Sdc4 and EGFR is direct, as purified recombinant ectodomains interact and are displaced from one another by SSTN_EGFR_. This peptide also blocks EGF-stimulated epithelial cell migration on LN332 that depends on α3β1 integrin but has no effect on HER2-stimulated migration. Like the juxtamembrane site in Sdc1, there are strong hints that the site in Sdc4 is also multifunctional. It was first described by McFall and Rapraeger as a site in Sdc4 that promoted the adhesion of fibroblasts and endothelial cells, although binding partners were not identified (98, 99). Further work from Couchman and Whiteford has shown that conserved amino acids within this site (I^89^ and the C-terminal GAT motif) regulate β1 integrin function in zebrafish, although potential interacting partners remain uncertain in those studies as well (100).

As with the other syndecan-organized signaling complexes, questions remain on how association with the syndecan and matrix-mediated clustering may serve to activate HER2 or EGFR and whether this occurs independent of classical growth factor binding. It is clear that stimulation of cell migration by EGFR paired with Sdc4 and the laminin-binding integrins still requires EGF (81, 83). This requirement for an RTK ligand contrasts with each of the other syndecan-organized complexes that have been described, including the HER2 mechanism, where clustering mediated by the adhesion receptors in the receptor complex appears to be sufficient to activate the associated RTK (IGF1R, HER2, VEGFR2) (41, 60, 81). It is possible that this traces to the specific mechanisms that regulate activation of the EGFR family of kinases. Activation of EGFR and HER2 kinases is known be caused by receptor dimerization, leading to head-to tail interactions of their cytoplasmic kinase domains that convert them to the active conformation (101). For EGFR, receptor dimerization is highly regulated by interactions in extracellular domains II and IV that are dependent on growth factor binding (102–104). EGF binding alters the conformation of domain IV, allowing the receptor to transition from a “closed” to an “open” conformation, relieving steric hindrance that otherwise prevents the adjacent receptors from dimerizing. In addition, EGF binding exposes a dimerization arm in domain II that interacts with a corresponding arm in the adjacent partner, stabilizes the receptor pair, and causes kinase activation through “head to tail” interactions of the two kinase domains (102–105) (**Figure 3C**). It is intriguing to note that HER2 is an orphan receptor that does not depend on ligand binding and is not inhibited by these regulatory sequences in domains II and IV (102–104). In its native state it favors the “open” conformation with exposed dimerization arm and as such does not depend on ligand binding in order to dock with other ligand-activated EGFR family members, such as EGF-bound EGFR (**Figure 3C**). This may be key to the mechanisms for HER2 and EGFR activation when assembled with the syndecan and integrins. HER2 is autoactivated when the quaternary Sdc1-HER2-integrin complex becomes clustered, either by integrin or syndecan-mediated binding to LN332, or when cells are plated solely on an artificial substratum consisting of β4-integrin-specific antibodies, suggesting that HER2 is captured by Sdc1 as a monomer, and becomes activated when the syndecan with which it is associated is clustered (81, 82). On the same cells, EGFR engaged with Sdc4, α3β1 integrin and α6β4 integrin is not activated when exposed to the same native LN332 or artificial β4-integrin antibody substrata unless EGF is provided. This suggests that matrix-driven clustering is sufficient to activate the kinases, as observed with HER2, but cannot act alone if impediments to dimerization require the additional action of a growth factor.

## Summary and Perspectives

The lack of amino acid conservation and recognizable structural motifs in syndecan extracellular domains across each of the four syndecan family members, in sharp contrast to shared motifs in their transmembrane and cytoplasmic domains, belies an important organizer role for these domains in which each syndecan functions as a highly specific organizer of RTKs and integrins at the cell-matrix interface. Each of the RTKs identified to date, EGFR, HER2, VEGFR2 and IGF-1R, have been well-studied and structurally characterized. Yet, their interactions with syndecans have only recently come to light, suggesting that further work may identify a similar dependence for other RTKs. The general picture that emerges is one where RTK signaling can be triggered either by the binding of soluble cytokines or growth factors to exert paracrine growth control over a population of cells, or by syndecans engaged with the ECM that directly pair the RTK with integrins, leading to autocrine, growth factor-independent RTK activation, cell migration and survival. These local mechanisms are likely to play a role in wound healing, where cells depend on the activation of a migratory apparatus and anti-apoptosis signaling as they migrate out of their normal niche and are likely to be co-opted by tumor cells that rely on the same mechanisms for metastasis and survival. Whereas organizer roles for Sdc1 and Sdc4 have now been described, similar roles for Sdc2 and Sdc3 remain unknown but seem likely to exist as well. The examples discovered to date show that a single syndecan may have multiple organizer activities, as revealed by three distinct organizer activities for Sdc1 alone. Sdc4 also appears to be multifunctional, although the full extent of its repertoire remains to be defined. The lack of structural data for any of the four syndecan extracellular domains hampers a full analysis of how these multifunctional sites work to assemble distinct sets of receptors but lead to the speculation that certain amino acids provide a docking structure or motif while others are critical for specific protein-protein interactions with their target receptors. SSTN peptides derived from these sites benefit from this high degree of specificity, which, together with the targets being extracellular, provide promising approaches to target tumor cells and tumor-induced angiogenesis. To date, such studies have relied on cell culture and animal models of tumorigenesis and angiogenesis (SSTN_IGF1R_). Further work involving all synstatins in animal models is ongoing and will hopefully be extended to human cancer in future clinical trials.

## Author Contributions

The author confirms being the sole contributor of this work and has approved it for publication.

## Funding

The work summarized in this review was supported by funds from the National Institutes of Health to the author’s laboratory (R01-CA139872, R01-CA118839, R01-CA163662, R01-DE028341), and to the University of Wisconsin Carbone Cancer Center (P30-CA014520).

## Conflict of Interest

The author declares that the research was conducted in the absence of any commercial or financial relationships that could be construed as a potential conflict of interest.

## Publisher’s Note

All claims expressed in this article are solely those of the authors and do not necessarily represent those of their affiliated organizations, or those of the publisher, the editors and the reviewers. Any product that may be evaluated in this article, or claim that may be made by its manufacturer, is not guaranteed or endorsed by the publisher.
